# Pediatric Diabetes Prevalence Among Medicaid Beneficiaries

**DOI:** 10.1001/jamanetworkopen.2025.60507

**Published:** 2026-02-23

**Authors:** Hao Zhang, Theodoros Giannouchos, David Becker, Ye Liu, Jillur Rahim, Pradeep Sharma, Ambika Ashraf, Barbara Gower, David Allison, Ananda Basu, Rita Basu, Julie McDougal, Bisakha Sen

**Affiliations:** 1 Department of Health Policy and Organization, University of Alabama at Birmingham, Birmingham; 2 Department of Pediatrics, University of Alabama at Birmingham, Birmingham; 3 Department of Nutrition Sciences, University of Alabama at Birmingham, Birmingham; 4 Children's Nutrition Research Center, Baylor College of Medicine, Houston, Texas; 5 Department of Medicine, University of Alabama at Birmingham, Birmingham

## Abstract

**Importance:**

Pediatric diabetes, an increasing public health concern in the US, is associated with lifelong health complications and substantial societal costs. The burden is particularly severe among socioeconomically at-risk populations. Medicaid and the Children’s Health Insurance Program (CHIP) insure 49% of all youths in the US, disproportionately covering socioeconomically at-risk populations. However, national data on prevalence and trends in pediatric diabetes among Medicaid and CHIP enrollees are lacking.

**Objectives:**

To provide comprehensive nationwide estimates and trends in the prevalence of type 1 diabetes (T1D) and type 2 diabetes (T2D) among Medicaid and CHIP pediatric enrollees from 2016 to 2021 and to assess variation across demographic and geographic subpopulations.

**Design, Setting, and Participants:**

This repeated cross-sectional study used 2016 to 2021 Transformed Medicaid Statistical Information System Analytical Files to study Medicaid and CHIP enrollees aged 18 years and younger with continuous full-year enrollment in each study year across 43 states (7 states and Washington, DC, were excluded due to unstable continuous enrollment proportions or other data quality issues). Trends were assessed using the Cochran-Armitage test.

**Exposures:**

Demographic (age, sex, race, and ethnicity) and geographic (census region and urban vs rural) characteristics

**Main Outcomes and Measures:**

Main outcomes were standardized annual prevalence of T1D and T2D per 1000 enrollees. Subgroup trends were stratified by age, sex, race and ethnicity, region, and urbanicity.

**Results:**

Approximately 25 million to 30 million youths were included in the study each year. Across all study years, enrollees aged 0 to 6 years and 7 to 12 years each accounted for approximately 35% of the Medicaid pediatric population (8 887 176 of 25 537 653 [34.9%] and 9 069 241 [35.6%] in 2016 and 10 025 946 [33.2%] and 10 308 534 of 30 324 022 [34.1%] in 2021, respectively), whereas those aged 13 to 18 years represented approximately 30% (7 484 682 [29.4%] in 2016 and 9 905 058 [32.8%] in 2021). The proportion of male enrollees was slightly higher than female enrollees (13 074 795 [51.2%] males vs 12 452 685 [48.8%] females in 2016 and 15 528 719 [51.2%] vs 14 794 520 [48.8%] in 2021). From 2016 to 2021, pediatric diabetes prevalence among Medicaid-enrolled youths increased from 2.73 to 3.04 per 1000 enrollees (11.4% relative increase; *P* < .001). T1D increased from 1.99 to 2.12 (6.5%) and T2D from 0.74 to 0.92 (24.3%). T2D increases were especially pronounced among males (48.1%) and residents of the western US (51.8%). Rural residents had a higher prevalence of diabetes across all study years but a substantially lower relative increase compared with urban residents (4.7% vs 13.3%).

**Conclusions and Relevance:**

In this cross-sectional study of Medicaid-enrolled youths, the prevalence of pediatric diabetes steadily increased, underscoring an increasing public health challenge. The sharp increase in T2D highlights the urgent need for targeted prevention, screening, and management strategies to mitigate long-term health and economic consequences.

## Key Points

**Questions:**

How has the prevalence of type 1 and type 2 diabetes changed from 2016 to 2021 within Medicaid- and Children’s Health Insurance Program–enrolled youths aged 18 years or younger across the US, and how have trends varied by demographic and geographic characteristics?

**Findings:**

In this cross-sectional study of 25 to 30 million youths across 43 states, pediatric diabetes prevalence increased by 11.4%, with type 2 diabetes increasing by 24.3%. The largest increases occurred among males, Hispanic youths, urban youths, and residents of the western US.

**Meaning:**

The increasing burden of pediatric diabetes among publicly insured youths, including historically lower-risk populations, highlights the urgent need for prevention, early screening, and targeted interventions.

## Teaser

This cross-sectional study examines changes in the prevalence of diabetes among youths aged 18 years or younger in 43 states from 2016 to 2021.

## Introduction

The prevalence of both type 1 diabetes (T1D) and type 2 diabetes (T2D) has increased substantially among the pediatric population in the US.^1^ T1D is an autoimmune condition with a genetic component that has historically accounted for the most cases of diabetes among youths.^2,3^ T2D is a multifactorial disease driven by both genetic and environmental factors, characterized by insulin resistance and β-cell dysfunction, and influenced by genetic predisposition, obesity, sedentary behavior, and poor diet.^4,5^ For either type of diabetes, effective lifelong management is essential.

Pediatric diabetes is associated with impaired physical fitness,^6,7^ poorer quality of life,^8,9,10^ and increased risk of premature morbidity.^11^ It is also associated with long-term complications, such as hypertension, premature cardiovascular disease, retinopathy, chronic kidney disease, and neuropathy.^10,12,13,14^ In addition, the burdens extend beyond youths, imposing substantial physical, emotional, and financial strain on parents and caregivers.^15,16,17^ Given its far-reaching health and social consequences, a deeper assessment of the prevalence of pediatric diabetes across regions and subpopulations is critical to inform disease management strategies.

There is evidence that, during the last 2 decades, the prevalence of pediatric diabetes in the US has been steadily increasing.^1,3,10^ Estimates from the multicenter SEARCH study based in 6 states and tribal lands reported that the age-, sex-, and race- and ethnicity-adjusted incidence rates of T1D and T2D increased by 2.0% and 5.3% per year, respectively, from 2002 through 2018.^18,19^ Youths from low-income families, racial and ethnic minority groups, and those residing in rural or medically underserved regions have an increased risk of developing pediatric diabetes.^3,20^ Socioeconomic deprivation, including food insecurity, poverty, inadequate recreational spaces, and limited access to health care services, exacerbate T2D risk,^21^ whereas increased exposure to infections and toxins may trigger T1D.^22^ Furthermore, youths from families with low-income may have limited access to resources for timely and comprehensive disease management,^23,24^ which can further increase the risk of severe diabetes complications.

Medicaid is the largest public health insurance program in the country and, together with the Children’s Health Insurance Program (CHIP), provides coverage to approximately 49% of the pediatric population, disproportionately covering socioeconomically at-risk populations.^25,26^ However, national estimates on the prevalence of pediatric diabetes among this population remain scarce. To date, we are aware of only one study^27^ that focused on Medicaid and analyzed the prevalence of pediatric diabetes between 2002 and 2016 using the Truven Health MarketScan Multi-State Medicaid Database, which includes Medicaid data from 11 geographically dispersed but deidentified states in the database.^28^ Given the rapid increase in diabetes incidence and prevalence in the past decade and concerns about exacerbation during the COVID-19 pandemic, updated and comprehensive estimates of pediatric diabetes among socioeconomically at-risk youths are needed to develop appropriate intervention strategies.

This study uses data that the Centers for Medicare & Medicaid Services has made available to researchers since 2019 and aims to offer the first comprehensive overview of pediatric diabetes prevalence among Medicaid-enrolled youth from 2016 to 2021 in the US. We also examined variation in disease burden by demographics and by region to identify potential targets for prevention and disease management interventions.

## Methods

### Data Source

This repeated cross-sectional study used the Transformed Medicaid Statistical Information System Analytical Files (TAFs) for 2016 to 2021 to analyze trends in pediatric diabetes prevalence among Medicaid and CHIP (hereafter referred to as Medicaid) beneficiaries. The TAF database includes detailed enrollment records, demographics, and medical and prescription drug claims for the entire Medicaid population across 50 states, the District of Columbia, and US territories. The TAF database enabled us to estimate the prevalence of diabetes among Medicaid-enrolled youths at the population level. The TAF data were further linked to the 2010 Rural-Urban Commuting Area Codes dataset^29^ to examine urban-rural differences. This study was approved by the University of Alabama at Birmingham institutional review board. Patient consent was waived by the institutional review board because this study involved secondary analysis of deidentified administrative claims data and posed minimal risk to humans. This study followed the Strengthening the Reporting of Observational Studies in Epidemiology (STROBE) reporting guideline for cross-sectional studies.

### Study Population

In each calendar year from 2016 to 2021, the study population included all enrollees aged 18 years and younger who were continuously enrolled in Medicaid during the entire calendar year without any coverage gaps. Coverage gaps were determined based on the number of days covered in Medicaid in each calendar month. This strict definition ensured full information on health care service use during the calendar year and minimized the probability of misclassification errors because of enrollees receiving a diabetes diagnosis or treatment when they were not enrolled in Medicaid. In sensitivity analyses, the continuous enrollment requirement was relaxed to allow up to a total of 14 days in coverage gaps throughout the calendar year. Enrollees from US territories were excluded because their Medicaid programs differ substantially from the 50 US states and the District of Columbia regarding eligibility criteria and program benefits.

We assessed the stability in the share of continuously enrolled population across the study period by state. Higher proportions of continuously enrolled youths were found in 2020 and 2021, which was expected due to the continuous coverage requirements implemented during the COVID-19 public health emergency.^30^ Data from 4 states (Arkansas, Tennessee, Rhode Island, and Wyoming) and the District of Columbia were excluded due to very high within-state volatility in continuous enrollment rates before the COVID-19 pandemic (an absolute change >20% between 2 consecutive years between 2016 and 2019). Among the remaining 46 states, 50% to 90% of beneficiaries met the continuous enrollment criterion annually (eTable 1 in Supplement 1). During 2020 to 2021, all states exhibited a substantial increase in continuous enrollment due to the Medicaid continuous enrollment provision mandated in the Families First Coronavirus Response Act of 2020^31^ and more families becoming Medicaid eligible due to pandemic-era income and employment loss.^32^ Florida, Maryland, and Mississippi were subsequently excluded due to outlier data points in the time series of diabetes prevalence identified in later steps, raising concerns about data quality (eTable 2 in Supplement 1).

### Identification of T1D and T2D

Based on existing literature,^27,33^ diabetes cases were identified in each calendar year if an individual had either (1) 1 medical claim (inpatient, outpatient, or physician) with an *International Classification of Diseases, Tenth Revision (ICD-10)* diagnosis code for diabetes and at least 2 prescription claims for antidiabetic medications or (2) at least 2 medical claims with *ICD-10* diagnosis codes for diabetes that were at least 30 days apart. Youths with *ICD-10* codes for secondary diabetes, diabetes insipidus, or gestational diabetes were excluded. Antidiabetic medications were identified using the National Drug Code and Healthcare Common Procedure Coding System.

For youths who met the diagnosis criteria for diabetes, we then used an existing algorithm from the literature to categorize them as having T1D or T2D.^27,33^ Specifically, youths were defined to be diagnosed with T1D if any of the following 3 criteria were met: (1) younger than 6 years at diagnosis, (2) only had claims for insulin and no other antidiabetic drugs, or (3) T1D-specific diagnosis codes on the first 2 diabetes claims. Those who met the diagnosis criteria for diabetes but did not meet the criteria for T1D were considered as diagnosed with T2D. The codes used for diabetes identification are provided in eTable 3 in Supplement 1.

### Statistical Analysis

Annual diabetes prevalence rates were calculated by dividing the number of diabetes cases by the total number of eligible, continuously enrolled beneficiaries per calendar year and standardizing the rates with respect to age, sex, race and ethnicity, census region, and urban vs rural residence. Temporal trends in standardized prevalence were visualized using a time series plot. We further examined the standardized prevalences separately by demographic and geographic factors, including age (0-6, 7-12, and 13-18 years), sex (male vs female), race and ethnicity (Asian, Hispanic [regardless of race], non-Hispanic Black, non-Hispanic White, and other [American Indian or Alaska Native, Hawaiian or Pacific Islander, multiracial, and other]), census regions (Midwest, Northeast, South, and West), and place of residence (urban vs rural). Race and ethnicity data were collected by state Medicaid agencies during the enrollment process. We examined prevalence of pediatric diabetes by race and ethnicity to examine potential racial differences in the disease burden. For each of the subgroup analyses, the standardization was with respect to the remaining covariates. The pooled study population across all study years served as the standard population. Pairwise deletion was used in the stratified analysis. Less than 1% of the individuals were excluded for missing covariate values in each stratified analysis with the exception of race and ethnicity, for which 18% were dropped due to the exclusion of states with high data quality concerns.

One well-known challenge with TAF data is the high rate of missing values for race and ethnicity in some states. To address this issue, we excluded states where more than 50% of enrollees had missing race and ethnicity data in a given calendar year from the subgroup analyses by race and ethnicity. This approach aligns with the Data Quality Atlas definition of states with unusable data quality^34^ (see eTable 4 in Supplement 1 for states excluded in each year).

We performed the Cochran-Armitage test to evaluate whether there was a statistically significant linear trend in the outcomes across calendar years. A sensitivity analysis relaxing the study population to include those with 14 days or less in Medicaid coverage gaps, although still excluding states with poor data quality, was conducted to assess the robustness of the study finding. Two-sided *P* < .05 was considered significant. All analyses were conducted using SAS, version 9.4 (SAS Institute Inc).

## Results

Our study population included approximately 25 million youths annually from 2016 to 2019 and increased to 28 million and 30 million in 2020 and 2021, respectively (Table 1). Across all study years, enrollees aged 0 to 6 years and 7 to 12 years each accounted for approximately 35% of the Medicaid pediatric population (8 887 176 of 25 537 653 [34.9%] and 9 069 241 of 30 324 022 [35.6%] in 2016 and 10 025 946 [33.2%] and 10 308 534 [34.1%] in 2021, respectively), whereas those aged 13 to 18 years represented approximately 30% (7 484 682 [29.4%] in 2016 and 9 905 058 [32.8%] in 2021). The proportion of male enrollees was slightly higher than female enrollees (13 074 795 [51.2%] males vs 12 452 685 [48.8%] females in 2016 and 15 528 719 [51.2%] vs 14 794 520 [48.8%] in 2021). Asian enrollees numbered 866 057 (4.1%) in 2016 and 991 255 (3.8%) in 2021, Hispanic enrollees numbered 6 859 451 (32.4%) in 2016 and 8 073 682 (31.0%) in 2021, non-Hispanic Black enrollees numbered 4 499 886 (21.2%) in 2016 and 5 567 366 (21.4%) in 2021, non-Hispanic White enrollees numbered 8 344 243 (39.4%) in 2016 and 10 275 947 (39.4%) in 2021, and enrollees of other race numbered 621 224 (2.9%) in 2016 and 1 154 737 (4.4%) in 2021. Geographically, approximately one-third of enrollees resided in the South, followed by the West, Midwest, and Northeast. Urban residents consistently made up more than 80% of the study sample.

**Table 1.** Demographic Characteristics of the Study Population by Year^a^

Characteristic^b^	No. (%) of population						*P* value
	2016 (n = 25 537 653)	2017 (n = 25 683 703)	2018 (n = 25 441 391)	2019 (n = 24 983 107)	2020 (n = 27 653 028)	2021 (n = 30 324 022)	
Age group, y							
0-6	8 887 176 (34.9)	8 836 105 (34.5)	8 676 073 (34.3)	8 404 489 (33.8)	9 279 463 (33.7)	10 025 946 (33.2)	<.001
7-12	9 069 241 (35.6)	9 113 680 (35.6)	8 989 248 (35.5)	8 763 916 (35.2)	9 504 420 (34.5)	10 308 534 (34.1)	
13-18	7 484 682 (29.4)	7 630 760 (29.8)	7 665 100 (30.3)	7 707 498 (31.0)	8 772 095 (31.8)	9 905 058 (32.8)	
Sex							
Male	13 074 795 (51.2)	13 155 397 (51.2)	13 034 464 (51.2)	12 806 001 (51.3)	14 172 157 (51.3)	15 528 719 (51.2)	<.001
Female	12 452 685 (48.8)	12 523 489 (48.8)	12 402 869 (48.8)	12 174 751 (48.7)	13 479 909 (48.7)	14 794 520 (48.8)	
Race and ethnicity^c^^,^^d^							
Asian	866 057 (4.1)	876 106 (4.3)	866 994 (4.3)	818 527 (4.0)	890 651(3.9)	991 255 (3.8)	<.001
Hispanic	6 859 451 (32.4)	6 834 702 (33.2)	6 622 953 (32.6)	6 304 712 (30.6)	6 860 093 (30.1)	8 073 682 (31.0)	
Non-Hispanic Black	4 499 886 (21.2)	4 293 659 (20.8)	4 271 436 (21.0)	4 463 161 (21.7)	5 024 174 (22.0)	5 567 366 (21.4)	
Non-Hispanic White	8 344 243 (39.4)	7 958 546 (38.6)	7 905 098 (38.9)	8 334 826 (40.5)	9 236 815 (40.5)	10 275 947 (39.4)	
Other	621 224 (2.9)	635 900 (3.1)	641 898 (3.2)	658 237 (3.2)	802 556 (3.5)	1 154 737 (4.4)	
Census region							
Midwest	5 693 372 (22.3)	5 705 543 (22.2)	5 551 887 (21.8)	5 349 986 (21.4)	6 000 728 (21.7)	6 666 950 (22.0)	<.001
Northeast	4 213 585 (16.5)	4 278 999 (16.7)	4 463 613 (17.5)	4 504 191 (18.0)	5 005 620 (18.1)	5 361 668 (17.7)	
South	7 956 039 (31.2)	8 199 246 (31.9)	8 120 917 (31.9)	8 035 456 (32.2)	9 063 484 (32.8)	10 134 916 (33.4)	
West	7 674 657 (30.1)	7 499 915 (29.2)	7 304 974 (28.7)	7 093 474 (28.4)	7 583 196 (27.4)	8 160 488 (26.9)	
Residence							
Urban	20 248 453 (82.3)	21 004 267 (82.4)	20 832 088 (82.4)	20 506 290 (82.4)	22 714 433 (82.4)	24 921 169 (82.5)	<.001
Rural	4 369 053 (17.7)	4 498 840 (17.6)	4 451 716 (17.6)	4 385 417 (17.6)	4 840 061 (17.6)	5 294 538 (17.5)	

^a^ Data from 5 states (Arkansas, Florida, Maryland, Rhode Island, and Tennessee) and Washington, DC, were excluded due to data quality issues.

^b^ Numbers for each variable may not add up to the total due to pairwise deletion.

^c^ The other group includes American Indian or Alaska Native, Hawaiian or Pacific Islander, multiracial, and other.

^d^ States with the missingness rate greater than 50% (including 6 states in 2016, 5 states in 2017, 3 states in 2018, 2 states in 2019, and 4 states in 2020) were excluded from calculation.

From 2016 to 2021, the prevalence of pediatric diabetes (T1D and T2D combined) among Medicaid-enrolled youths increased steadily from 2.73 to 3.04 per 1000 enrollees (Figure), an 11.4% relative increase (*P *for trend < .001). When examined by diabetes type, the prevalence of T1D increased from 1.99 to 2.12 per 1000 enrollees (relative increase, 6.5%; *P* < .001), whereas the prevalence of T2D increased from 0.74 to 0.92 per 1000 enrollees (relative increase, 24.3%; *P* < .001) (Figure). Crude prevalences are presented in the eFigure in Supplement 1.

**Figure.**
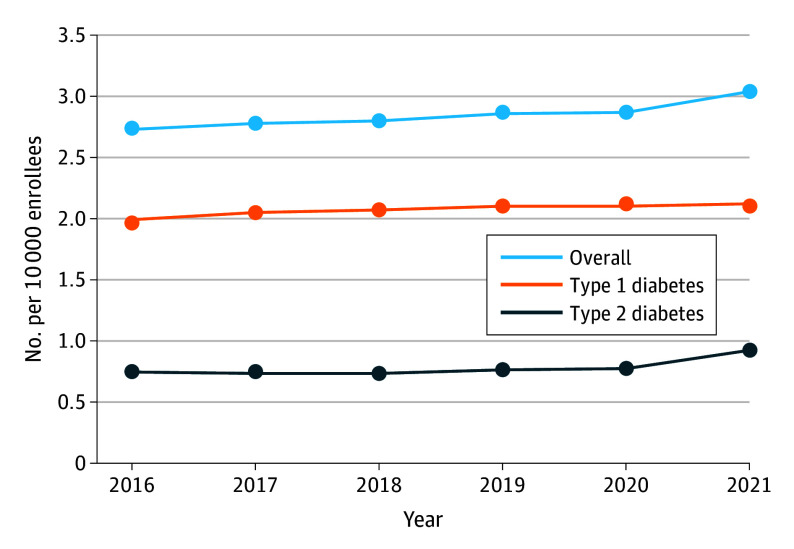
Trends in the Standardized Prevalence of Pediatric Diabetes Among Medicaid and Children’s Health Insurance Program Enrollees, 2016 to 2021

Analysis by demographic and geographic variables revealed significant differences in both prevalence and temporal trends across population subgroups (Table 2). Among distinct age groups, those aged 13 to 18 years had the highest prevalence compared with the other age groups of 7 to 12 years and 0 to 6 years. However, the 7- to 12-year age group exhibited the largest relative increase over time of 14.1% compared with 10.9% for the 0- to 6-year age group and 9.9% for the 13- to 18-year age group. Males had a lower prevalence compared with females but a larger relative increase (16.8% vs 6.4%). Non-Hispanic White enrollees had the highest prevalence across all years but the lowest relative increase (1.5%) compared with other racial and ethnic groups. In contrast, Hispanic, non-Hispanic Black, and other race and ethnic groups experienced large relative increases exceeding 20%. The West census region experienced a substantially higher relative increase compared with other census regions (22.8% compared with 7.7% for Midwest, 9.2% for Northeast, and 6.7% for South). Rural residents had a higher prevalence of diabetes across all study years but a substantially lower relative increase compared with urban residents (4.7% vs 13.3%).

**Table 2.** Standardized Prevalence of Pediatric Diabetes per 1000 Medicaid-Enrolled Youths by Demographic Characteristics^a^

Characteristic^b^	Prevalence per 1000 youths						Relative increase, %	*P* value^c^
	2016	2017	2018	2019	2020	2021		
Overall	2.73	2.78	2.80	2.86	2.87	3.04	11.4	<.001
Age group, y								
0-6	0.46	0.47	0.48	0.50	0.50	0.51	10.9	<.001
7-12	2.13	2.17	2.19	2.28	2.32	2.43	14.1	<.001
13-18	5.95	6.04	6.07	6.15	6.13	6.54	9.9	<.001
Sex								
Male	2.50	2.58	2.61	2.68	2.74	2.92	16.8	<.001
Female	2.97	2.99	2.99	3.05	3.01	3.16	6.4	<.001
Race and ethnicity^d^								
Asian	1.46	1.47	1.45	1.51	1.54	1.77	21.2	<.001
Hispanic	2.11	2.18	2.26	2.36	2.32	2.58	22.3	<.001
Non-Hispanic Black	2.73	2.79	2.83	2.94	3.05	3.5	28.2	<.001
Non-Hispanic White	3.43	3.46	3.44	3.5	3.43	3.48	1.5	<.001
Other^e^	2.39	2.53	2.55	2.62	2.70	3.07	28.5	<.001
Census region								
Midwest	2.98	3.07	3.12	3.23	3.07	3.21	7.7	<.001
Northeast	3.05	2.99	2.96	2.98	3.14	3.33	9.2	<.001
South	2.83	2.80	2.77	2.81	2.84	3.02	6.7	<.001
West	2.24	2.39	2.48	2.57	2.58	2.75	22.8	<.001
Residence								
Urban	2.63	2.68	2.71	2.76	2.79	2.98	13.3	<.001
Rural	3.18	3.23	3.21	3.27	3.23	3.33	4.7	<.001

^a^ Diabetes cases were identified if there were (1) 1 diabetes diagnosis and 2 antidiabetic drugs or (2) 2 diabetes diagnoses at least 30 days apart. The prevalence estimates were standardized with respect to age, sex, race, census region, and urban vs rural residence. For each covariate of interest, prevalence rates were standardized to the distribution of the remaining covariates using the pooled study population across all study years as the standard population. Data from 7 states (Arkansas, Florida, Mississippi, Maryland, Rhode Island, Tennessee, and Wyoming) and Washington, DC, were excluded due to data quality issues.

^b^ Pairwise deletion is used in the subgroup analysis.

^c^ *P* value from Cochran-Armitage trend test.

^d^ States with the missingness rate greater than 50% (including 5 states in 2016, 5 states in 2017, 3 states in 2018, 2 states in 2019, 4 states in 2020, and 1 state in 2021) were excluded from calculation.

^e^ The other group includes American Indian or Alaska Native, Hawaiian or Pacific Islander, multiracial, and other.

When examining T1D and T2D separately (Table 3 and Table 4), males and females had a similar prevalence estimate of T1D, but females had a higher prevalence of T2D (1.08 vs 0.77 per 1000 enrollees in 2021). However, the relative increase in T2D was substantially larger for males compared with females (48.1% vs 11.3%). Despite the highest overall prevalence of pediatric diabetes among non-Hispanic White enrollees, they had the lowest prevalence of T2D among all race and ethnicity groups as well as the smallest relative increase (relative increase, 1.8%; *P *for trend = .43). This contrasts sharply with other race and ethnicity groups, all of whom experienced substantial increases in T2D prevalence during the study period (41.5% for Asian, 40.0% for Hispanic, 41.2% for non-Hispanic Black, and 37.2% for other). The West census region exhibited the largest relative increases for both T1D (13.7%) and T2D (51.8%) among all census regions. Although the South had the second lowest overall prevalence of pediatric diabetes (2.83 to 3.02 per 1000 enrollees), its prevalence of T2D was the highest across all years (0.95 to 1.07 per 1000 enrollees). Rural residents exhibited higher prevalences of both T1D and T2D but experienced a lower relative increase compared with urban residents. The results from the sensitivity analysis, which included youths with Medicaid coverage gaps of no more than 14 days, were consistent with the main findings (eTables 5-7 in Supplement 1).

**Table 3.** Standardized Prevalence of Type 1 Diabetes per 1000 Medicaid-Enrolled Youths by Demographic Characteristics^a^

Characteristic^b^	Prevalence per 1000 youths						Relative increase, %	*P* value^c^
	2016	2017	2018	2019	2020	2021		
Overall	1.99	2.05	2.07	2.10	2.10	2.12	6.5	<.001
Age group, y								
0-6	0.46	0.47	0.48	0.50	0.50	0.51	10.9	<.001
7-12	1.85	1.90	1.93	1.99	2.01	2.05	10.8	<.001
13-18	3.87	3.97	4.00	4.01	4.00	4.00	3.4	<.001
Sex								
Male	1.99	2.05	2.07	2.10	2.13	2.15	8.0	<.001
Female	2.00	2.05	2.07	2.11	2.08	2.08	4.0	<.001
Race and ethnicity^d^								
Asian	0.93	0.92	0.92	0.95	0.95	1.02	9.7	<.001
Hispanic	1.31	1.37	1.43	1.46	1.42	1.46	11.5	<.001
Non-Hispanic Black	1.71	1.78	1.83	1.90	1.95	2.06	20.5	<.001
Non-Hispanic White	2.88	2.94	2.93	2.97	2.94	2.92	1.4	<.001
Other^e^	1.46	1.42	1.46	1.58	1.57	1.78	21.9	<.001
Census region								
Midwest	2.27	2.37	2.41	2.47	2.36	2.36	4.0	<.001
Northeast	2.39	2.37	2.34	2.33	2.47	2.47	3.3	<.001
South	1.87	1.88	1.88	1.92	1.93	1.95	4.3	<.001
West	1.68	1.78	1.85	1.89	1.88	1.91	13.7	<.001
Residence								
Urban	1.92	1.97	1.99	2.02	2.04	2.06	7.3	<.001
Rural	2.33	2.40	2.41	2.44	2.42	2.41	3.4	<.001

^a^ Type 1 diabetes was defined if any of the following were met: (1) younger than 6 years at diagnosis, (2) no antidiabetic drug use except for insulin, or (3) type 1 diabetes as the first 2 diagnoses. The prevalence estimates were standardized with respect to age, sex, race, census region, and urban vs rural residence. For each covariate of interest, prevalence rates were standardized to the distribution of the remaining covariates using the pooled study population across all study years as the standard population. Data from 7 states (Arkansas, Florida, Mississippi, Maryland, Rhode Island, Tennessee, and Wyoming) and Washington, DC, were excluded due to data quality issues.

^b^ Pairwise deletion is used in the subgroup analysis.

^c^ *P* value from Cochran-Armitage trend test.

^d^ States with the missingness rate greater than 50% (including 5 states in 2016, 5 states in 2017, 3 states in 2018, 2 states in 2019, 4 states in 2020, and 1 state in 2021) were excluded from calculation.

^e^ The other group includes American Indian or Alaska Native, Hawaiian or Pacific Islander, multiracial, and other.

**Table 4.** Standardized Prevalence of Type 2 Diabetes per 1000 Medicaid-Enrolled Youths by Demographic Characteristics^a^

Characteristic^b^	Prevalence per 1000 youths						Relative increase, %	*P* value^c^
	2016	2017	2018	2019	2020	2021		
Overall	0.74	0.73	0.73	0.76	0.77	0.92	24.3	<.001
Age group, y^d^								
0-6	NA	NA	NA	NA	NA	NA		
7-12	0.28	0.26	0.26	0.29	0.31	0.39	39.3	<.001
13-18	2.08	2.07	2.07	2.14	2.14	2.55	22.6	<.001
Sex								
Male	0.52	0.53	0.54	0.58	0.61	0.77	48.1	<.001
Female	0.97	0.94	0.93	0.95	0.93	1.08	11.3	<.001
Race and ethnicity^e^								
Asian	0.53	0.54	0.53	0.57	0.59	0.75	41.5	<.001
Hispanic	0.80	0.81	0.83	0.90	0.90	1.12	40.0	<.001
Non-Hispanic Black	1.02	1.01	1.00	1.04	1.09	1.44	41.2	<.001
Non-Hispanic White	0.55	0.52	0.51	0.53	0.50	0.56	1.8	.43
Other^f^	0.94	1.11	1.09	1.04	1.14	1.29	37.2	<.001
Census region								
Midwest	0.71	0.70	0.70	0.76	0.71	0.84	18.3	<.001
Northeast	0.66	0.62	0.63	0.65	0.68	0.85	28.8	<.001
South	0.95	0.92	0.88	0.89	0.91	1.07	12.6	<.001
West	0.56	0.61	0.63	0.68	0.70	0.85	51.8	<.001
Residence								
Urban	0.71	0.71	0.71	0.74	0.75	0.92	29.6	<.001
Rural	0.85	0.83	0.8	0.83	0.81	0.92	8.2	<.001

Abbreviation: NA, not applicable.

^a^ Type 2 diabetes was defined if none of the criteria for type 1 diabetes was met. The prevalence estimates were standardized with respect to age, sex, race, census region, and urban vs rural residence. For each covariate of interest, prevalence rates were standardized to the distribution of the remaining covariates using the pooled study population across all study years as the standard population. Data from 7 states (Arkansas, Florida, Mississippi, Maryland, Rhode Island, Tennessee, and Wyoming) and Washington, DC, were excluded due to data quality issues.

^b^ Pairwise deletion is used in the subgroup analysis.

^c^ *P* value from Cochran-Armitage trend test.

^d^ Pediatric diabetes for youths aged 0 to 6 years were classified as type 1 based on our algorithm.

^e^ States with the missingness rate greater than 50% (including 5 states in 2016, 5 states in 2017, 3 states in 2018, 2 states in 2019, 4 states in 2020, and 1 state in 2021) were excluded from calculation.

^f^ The other group includes American Indian or Alaska Native, Hawaiian or Pacific Islander, multiracial, and other.

## Discussion

Despite Medicaid being the single largest source of health insurance coverage for the pediatric population in the US, comprehensive documentation on the prevalence of pediatric diabetes among Medicaid enrollees has been scarce. This is the first study, to our knowledge, to document prevalence and trends in diabetes among pediatric Medicaid beneficiaries across the US by leveraging TAF data from the Centers for Medicare & Medicaid Services. Our study found that the prevalence of diabetes increased annually from 2.73 to 3.04 per 1000 enrollees between 2016 and 2021 among continuously enrolled Medicaid youths. The prevalence of T1D increased from 1.99 to 2.12 per 1000 enrollees, and the prevalence of T2D increased from 0.74 to 0.92 per 1000 enrollees. Additionally, we found that both prevalence and trends in pediatric diabetes varied noticeably by demographics, geographic region, and urbanicity, particularly for T2D.

Precise magnitude comparisons are infeasible due to differences in study design, population and time frame; however, broad similarities and differences between our findings and those of earlier studies are noted.^1,10,27,33^ One study focusing on a nationwide, commercially insured population reported that T1D prevalence increased from 1.48 to 2.32 per 1000 youths and T2D from 0.38 to 0.49 per 1000 youths from 2002 to 2013.^33^ In contrast, a study using the MarketScan Multi-State Medicaid data from 11 unidentified states found a decrease in T2D prevalence between 2011 and 2016.^27^ The landmark SEARCH study^1^ that used data from 6 areas of the US estimated that, between 2001 and 2017, pediatric T1D prevalence increased from 1.48 to 2.15 per 1000 youths and T2D from 0.34 to 0.67 per 1000 youths for relative increases of 45.1% and 95.3%, respectively, during these 16 years. Our study, which started in 2016, found that the prevalence of T1D and T2D in the Medicaid-enrolled pediatric populations has increased steadily from 2016 to 2021, with relative increases of 6.5% for T1D and 24.3% for T2D in these 5 years.

One potentially unique contribution of this study was the assessment of variations in pediatric diabetes by census region. The South census region had the highest T2D prevalence in the nation relative to other regions, potentially attributed to a variety of sociodemographic, economic, and policy factors.^35^ Moreover, states that did not expand Medicaid under the Patient Protection and Affordable Care Act are predominantly in the South census region,^36^ and spillover effects on youths of more stringent eligibility requirements for adults have been documented.^37^ At the same time, the West census region exhibited the sharpest increase in both T2D prevalence (by 51.8%) and T1D prevalence (by 13.7%) in our study period, which warrants attention. Similarly, although the prevalences of T1D and T2D were higher among the rural and female enrollees, the increases in prevalence were substantially higher among the urban residents and males. Notably, T2D prevalence was lowest among non-Hispanic White enrollees and demonstrated a smaller relative increase during our study period compared with all other racial and ethnic groups, suggesting that, even within the Medicaid population, race and ethnicity differences in lifestyle behaviors, access to healthy foods, and environmental stressors may exist.^38,39,40,41^ Of note, these race and ethnicity differences in T2D prevalence changes are broadly similar findings in the SEARCH study from 2001 to 2017.^1^ In contrast, whereas SEARCH found that non-Hispanic White youth had the largest increase in T1D prevalence from 2001 to 2017, we found that non-Hispanic White Medicaid enrollees had a smaller increase in T1D prevalence from 2016 to 2021 compared with all other racial and ethnic groups. Lastly, as previously mentioned, we noted a substantially higher relative increase in prevalence for males compared with females for T1D and T2D. SEARCH results indicated that for both T1D and T2D, from 2001 to 2009 the prevalence increased more sharply for females than males, but the pattern shifted from 2009 to 2017 and the percentage increase in prevalence for males crept above the percentage increase in prevalence for females.

Beyond the general trend of an increase in diabetes prevalence during our study period, we noted a sharp increase in 2021, primarily driven by T2D. This finding aligns with several previous studies^42,43,44,45^ reporting a surge in new-onset pediatric T2D diagnosis during the COVID-19 pandemic and increasing evidence that the COVID-19 infection is associated with exacerbated risk of T2D, although the mechanistic pathways are still being explored.^43,46,47^ Increased psychological stress,^48^ physical inactivity, and exacerbated risk of obesity due to shutdowns and other pandemic-era disruptions may have also contributed.^49^ Lastly, pandemic-era economic disruptions brought families into Medicaid who previously had higher incomes or employer-sponsored insurance, and their health care–seeking behaviors, including seeking diabetes screening for their children, may have differed from prepandemic Medicaid enrollees.^50,51^

### Limitations

We acknowledge several limitations. The claims-based algorithm we used to identify diabetes diagnoses has been used in previous studies with pediatric Medicaid enrollees^27,33^ but has not been formally validated, although claims-based algorithms have shown acceptable accuracy in other pediatric subpopulations.^52^ However, to our knowledge, no validated algorithm specific to Medicaid enrollees currently exists, and algorithms developed using other subpopulations, especially those that require 2 or 3 diagnoses within a limited period, are unlikely to be appropriate for Medicaid enrollees given the continuity of care challenges. Hence, we opted to use the algorithm in existing literature for Medicaid enrollees, which permits comparing our results to existing studies. Certain limitations are inherent in the TAF data, such as incomplete availability before 2016, an approximate 3-year lag before data become available to researchers, and high rates of missing race and ethnicity data for some states. Lastly, we recognize that rates for the past 2 years of our study (2020 and 2021) may be impacted by the unique circumstances of the COVID-19 pandemic, with evidence of spikes in new-onset T2D among publicly insured youth,^42,53,54^ an increase in the number of pediatric Medicaid enrollees due to the continuous enrollment policies under the COVID-19 public health emergency, and economic disruptions that drove more families to depend on public insurance. It is unclear how this combination of changes influenced the pediatric diabetes rates in 2020 to 2021.^55,56^

## Conclusions

This repeated cross-sectional study, to our knowledge, provides the first comprehensive estimates on prevalence and trends in pediatric diabetes among the Medicaid population across the US. We found an upward trend for both T1D and T2D among this population. Substantial variation in diabetes prevalence and changes in prevalence was found across demographic subgroups, geographic regions, and urbanicity. This study highlighted sharp increases in prevalence among subgroups and regions previously considered to be less at risk, namely, males, urban populations, and residents of the West census region. The findings can help guide resource allocation and public health efforts for prevention, screening, and management of pediatric diabetes.

## Notes

Supplement 1. **eTable 1.** Percentage of Medicaid-Enrolled Youth With 12-Month Continuous Enrollment by State and Year **eTable 2.** Prevalence of Pediatric Diabetes (per 1,000 Enrollees) Among Medicaid-Enrolled Youth, by State and Year **eTable 3.** Coding Algorithms for Pediatric Diabetes Identification Using ICD-10-CM, HCPCS, and NDC Codes in Medicaid Claims **eTable 4.** Proportion of Study Sample with Missing Race Information, by State and Year **eTable 5.** Standardized Prevalence of Pediatric Diabetes per 1,000 Medicaid-Enrolled Youths by Demographic Characteristics (Allowing up to a 14-Day Coverage Gap) **eTable 6.** Standardized Prevalence of Type 1 Diabetes per 1,000 Medicaid-Enrolled Youths by Demographic Characteristics (Allowing up to a 14-Day Coverage Gap) **eTable 7.** Standardized Prevalence of Type 2 Diabetes per 1,000 Medicaid-Enrolled Youths by Demographic Characteristics (Allowing up to a 14-Day Coverage Gap) **eFigure.** Trend in the Crude Prevalence of Pediatric Diabetes Among Medicaid and Children’s Health Insurance Program (CHIP) Enrollees, 2016 -2021

Supplement 2.
Data Sharing Statement
