# Supplementary material for: Pediatric Diabetes Prevalence Among Medicaid Beneficiaries
Source: JAMA Netw Open. 2026 Feb 23;9(2):e2560507. doi: 10.1001/jamanetworkopen.2025.60507 (PMC12931460; doi:10.1001/jamanetworkopen.2025.60507)
Supplement: Supplement 1. — eTable 1. Percentage of Medicaid-Enrolled Youth With 12-Month Continuous Enrollment by State and Year eTable 2. Prevalence of Pediatric Diabetes (per 1,000 Enrollees) Among Medicaid-Enrolled Youth, by State and Year eTable 3. Coding Algorithms for Pediatric Diabetes Identification Using ICD-10-CM, HCPCS, and NDC Codes in Medicaid Claims eTable 4. Proportion of Study Sample with Missing Race Information, by State and Year eTable 5. Standardized Prevalence of Pediatric Diabetes per 1,000 Medicaid-Enrolled Youths by Demographic Characteristics (Allowing up to a 14-Day Coverage Gap) eTable 6. Standardized Prevalence of Type 1 Diabetes per 1,000 Medicaid-Enrolled Youths by Demographic Characteristics (Allowing up to a 14-Day Coverage Gap) eTable 7. Standardized Prevalence of Type 2 Diabetes per 1,000 Medicaid-Enrolled Youths by Demographic Characteristics (Allowing up to a 14-Day Coverage Gap) eFigure. Trend in the Crude Prevalence of Pediatric Diabetes Among Medicaid and Children’s Health Insurance Program (CHIP) Enrollees, 2016 -2021 [file jamanetwopen-e2560507-s001.pdf]

Supplemental Online Content

Zhang H, Giannouchos T, Becker D, et al. Pediatric diabetes prevalence among Medicaid beneficiaries. *JAMA Netw Open*. 2026;9(2):e2560507. doi:10.1001/jamanetworkopen.2025.60507

eTable 1. Percentage of Medicaid-Enrolled Youth With 12-Month Continuous Enrollment by State and Year

eTable 2. Prevalence of Pediatric Diabetes (per 1,000 Enrollees) Among Medicaid-Enrolled Youth, by State and Year

eTable 3. Coding Algorithms for Pediatric Diabetes Identification Using ICD-10-CM, HCPCS, and NDC Codes in Medicaid Claims

eTable 4. Proportion of Study Sample with Missing Race Information, by State and Year

eTable 5. Standardized Prevalence of Pediatric Diabetes per 1,000 Medicaid-Enrolled Youths by Demographic Characteristics (Allowing up to a 14-Day Coverage Gap)

eTable 6. Standardized Prevalence of Type 1 Diabetes per 1,000 Medicaid-Enrolled Youths by Demographic Characteristics (Allowing up to a 14-Day Coverage Gap)

eTable 7. Standardized Prevalence of Type 2 Diabetes per 1,000 Medicaid-Enrolled Youths by Demographic Characteristics (Allowing up to a 14-Day Coverage Gap)

eFigure. Trend in the Crude Prevalence of Pediatric Diabetes Among Medicaid and Children's Health Insurance Program (CHIP) Enrollees, 2016 -2021

This supplemental material has been provided by the authors to give readers additional information about their work.

© 2026 Zhang H et al. *JAMA Network Open*.

eTable 1. Percentage of Medicaid-Enrolled Youth with 12-Month Continuous Enrollment by State and Year[a]

State	2016	2017	2018	2019	2020	2021
AK	75.4%	80.1%	82.3%	82.7%	85.1%	90.3%
AL	55.4%	52.3%	45.3%	48.2%	81.0%	88.7%
**AR***	43.5%	46.9%	75.5%	75.9%	85.7%	88.6%
AZ	78.7%	72.0%	69.7%	72.0%	82.8%	89.9%
CA	78.5%	78.4%	79.0%	78.9%	85.8%	89.6%
CO	80.4%	76.4%	72.0%	72.2%	82.9%	88.9%
CT	74.1%	73.9%	77.5%	78.6%	87.1%	89.4%
**DC***	46.4%	82.7%	83.9%	83.9%	89.4%	93.2%
DE	66.1%	72.4%	72.4%	71.7%	85.4%	87.0%
FL	84.7%	84.9%	84.1%	84.7%	90.3%	92.0%
GA	62.8%	68.0%	67.5%	72.1%	83.8%	90.1%
HI	82.4%	73.5%	71.3%	71.7%	80.7%	88.2%
IA	71.8%	72.2%	75.6%	77.6%	85.9%	90.0%
ID	81.5%	79.7%	77.2%	74.0%	81.8%	87.0%
IL	77.4%	78.6%	76.3%	76.7%	83.9%	88.9%
IN	72.8%	74.9%	72.9%	75.4%	85.0%	89.3%
KS	76.6%	70.0%	72.0%	70.1%	82.5%	88.2%
KY	86.9%	88.2%	87.6%	87.4%	88.4%	89.5%
LA	84.9%	83.7%	84.0%	79.8%	87.2%	92.2%
MA	69.1%	70.0%	69.0%	69.0%	81.1%	87.4%
MD	75.1%	77.6%	77.3%	78.5%	88.7%	91.3%
ME	73.3%	72.8%	71.9%	71.7%	78.0%	88.6%
MI	62.7%	74.0%	74.6%	72.5%	84.1%	89.9%
MN	68.9%	69.7%	67.8%	62.5%	77.8%	82.2%
MO	82.8%	80.0%	70.7%	62.6%	78.8%	88.6%
MS	79.9%	79.5%	73.3%	75.4%	87.6%	84.5%
MT	75.7%	81.4%	80.9%	72.5%	81.7%	90.9%
NC	82.7%	82.5%	82.0%	80.8%	88.2%	90.8%
ND	55.4%	58.2%	59.2%	57.7%	72.6%	80.4%
NE	67.5%	69.1%	69.5%	69.6%	78.3%	85.7%
NH	68.4%	65.5%	66.8%	66.6%	79.2%	87.9%
NJ	71.1%	71.2%	71.2%	70.4%	84.1%	89.7%
NM	84.7%	78.9%	76.5%	79.2%	88.6%	91.7%
NV	63.9%	65.5%	65.4%	64.0%	77.0%	85.2%
NY	63.2%	62.0%	64.9%	68.1%	84.7%	90.7%
OH	74.7%	74.2%	72.5%	71.9%	82.8%	89.4%
OK	62.0%	61.0%	60.8%	59.7%	78.7%	87.8%
OR	66.6%	69.3%	72.1%	73.0%	84.3%	88.0%
PA	49.1%	48.3%	52.6%	55.7%	78.3%	80.1%
**RI***	82.0%	83.9%	19.1%	24.1%	87.3%	90.3%

© 2026 Zhang H et al. *JAMA Network Open*.

SC	79.3%	77.4%	76.2%	79.2%	87.5%	91.7%
SD	66.9%	68.5%	67.6%	67.9%	81.5%	88.4%
**TN***	84.3%	60.1%	51.6%	57.2%	85.0%	88.0%
TX	62.2%	65.2%	64.2%	62.9%	77.9%	86.8%
UT	56.2%	55.0%	55.8%	55.0%	72.9%	82.3%
VA	71.5%	75.5%	77.7%	77.3%	88.3%	89.7%
VT	76.7%	79.7%	80.9%	79.2%	88.4%	91.8%
WA	80.6%	79.9%	79.4%	79.4%	85.6%	89.2%
WI	65.7%	56.6%	68.0%	68.8%	82.8%	90.3%
WV	71.5%	72.6%	71.3%	71.1%	82.0%	88.8%
**WY***	79.5%	59.0%	59.0%	58.5%	76.2%	85.5%

[a] States marked with (*) had an absolute change greater than 20% between two consecutive year between 2016 to 2019, and were excluded from analyses.

© 2026 Zhang H et al. *JAMA Network Open*.

eTable 2. Prevalence of Pediatric Diabetes (per 1,000 Enrollees) Among Medicaid-Enrolled Youth, by State and Year[a]

State	2016	2017	2018	2019	2020	2021
AK	1.73	1.73	1.94	1.79	1.99	2.22
AL	3.84	3.82	3.12	2.80	4.05	4.21
AZ	2.26	2.48	2.62	2.84	2.92	3.20
CA	1.97	2.07	2.14	2.25	2.33	2.65
CO	2.40	2.60	3.01	3.08	3.25	3.12
CT	3.39	3.26	3.19	3.24	3.28	3.37
DE	2.66	2.77	2.81	3.15	3.36	3.87
**FL***	2.11	1.64	0.87	2.18	2.26	2.51
GA	2.78	2.80	2.76	2.80	2.81	2.94
HI	1.47	1.76	1.72	1.69	1.83	2.29
IA	2.92	2.99	2.98	3.12	3.05	3.24
ID	2.81	3.10	3.27	3.55	3.70	3.79
IL	2.48	2.53	2.61	2.69	2.66	2.99
IN	2.64	2.60	2.65	2.68	2.75	2.84
KS	2.86	3.24	3.38	3.72	3.46	3.57
KY	3.14	2.91	3.03	3.01	3.00	3.36
LA	2.30	2.42	2.49	2.70	2.52	2.79
MA	2.83	2.86	2.97	2.95	2.93	3.29
**MD***	0.11	1.62	2.67	2.70	2.77	3.01
ME	3.48	3.72	3.99	3.80	3.51	3.56
MI	3.52	3.50	3.52	3.47	3.34	3.49
MN	3.16	3.40	3.45	3.86	3.73	4.06
MO	2.65	2.64	3.01	3.33	3.10	3.16
**MS***	2.77	3.27	3.60	3.66	3.62	0.08
MT	3.17	3.38	3.26	3.54	3.73	3.68
NC	2.72	2.80	2.86	2.98	3.09	3.33
ND	3.09	2.64	2.99	2.96	3.10	2.95
NE	2.79	2.80	2.80	3.01	2.90	3.12
NH	3.36	3.31	3.30	3.41	3.41	3.45
NJ	2.10	2.08	2.12	2.26	2.36	2.77
NM	2.11	2.26	2.42	2.48	2.63	2.92
NV	2.84	2.78	2.74	2.92	2.91	3.18
NY	2.22	2.13	2.05	2.07	2.35	2.63
OH	3.50	3.50	3.57	3.66	3.52	3.68
OK	2.59	2.66	2.55	2.58	2.59	2.66
OR	3.04	3.09	3.19	3.25	3.03	3.14
PA	5.79	5.87	5.72	5.67	5.27	5.56
SC	3.31	3.35	3.27	3.25	3.38	3.47
SD	3.23	3.51	3.59	3.95	3.69	3.66
TX	2.32	2.30	2.40	2.52	2.48	2.70

© 2026 Zhang H et al. *JAMA Network Open*.

UT	2.15	3.17	3.34	3.72	3.43	3.09
VA	3.26	3.11	3.01	3.03	3.11	3.34
VT	3.46	3.62	3.61	3.69	3.68	3.66
WA	2.61	2.64	2.76	2.93	2.94	3.17
WI	3.17	3.03	3.22	3.38	3.06	3.16
WV	3.77	3.86	3.76	3.74	3.58	3.65

[a] States marked with (*) had outliers in the time series (defined as relative changes in diabetes prevalence over 80% between both pairs of adjacent time points), and were excluded from analyses.

© 2026 Zhang H et al. *JAMA Network Open*.

eTable 3. Coding Algorithms for Pediatric Diabetes Identification Using ICD-10-CM, HCPCS, and NDC Codes in Medicaid Claims

Category	Codes
Type 1 diebetes ICD10 code	E10.x
Type 2 diebetes ICD10 code	E11.x
Secondary diebetes ICD10 code	E08.x, E09.x, E23.2, O24.4x
NDC codes for antidiabetic drugs (non-insulin)	Available upon request to the corresponding author
NDC codes for antidiabetic drugs (insulin)	Available upon request to the corresponding author
HCPCS codes for insulin	J1815, J1817, S5552, S5553, S5561, S5551, S5550, S5571, S5560, J1820, S5565, S5570, S5566, K0548

© 2026 Zhang H et al. *JAMA Network Open*.

eTable 4. Proportion of Study Sample with Missing Race Information, by State and Year[a]

State	2016	2017	2018	2019	2020	2021
AK	5.1%	5.4%	5.9%	6.2%	6.3%	6.5%
AL	**74.2%***	**76.9%***	**74.9%***	**71.0%***	**69.2%***	22.8%
AZ	32.9%	33.3%	33.7%	34.1%	34.3%	18.8%
CA	7.0%	7.0%	7.2%	7.6%	8.2%	8.8%
CO	**51.3%***	**52.6%***	46.6%	45.6%	45.7%	0.6%
CT	40.7%	42.2%	44.2%	45.8%	31.2%	31.0%
DE	0.0%	0.0%	0.0%	0.0%	0.0%	0.0%
GA	8.5%	8.4%	9.1%	10.2%	5.2%	6.9%
HI	18.5%	21.3%	26.0%	30.6%	35.1%	37.9%
IA	34.2%	31.0%	33.5%	33.6%	37.3%	37.2%
ID	0.0%	0.0%	0.0%	0.0%	0.0%	0.0%
IL	4.5%	4.0%	3.9%	4.8%	6.7%	9.7%
IN	3.3%	9.6%	10.2%	10.6%	16.0%	17.5%
KS	**70.7%***	**73.4%***	**73.4%***	**74.0%***	**77.2%***	9.4%
KY	13.2%	13.3%	13.5%	13.8%	14.1%	11.0%
LA	23.8%	28.4%	30.0%	32.1%	7.9%	8.1%
MA	48.6%	48.6%	49.0%	49.9%	46.8%	46.3%
ME	10.1%	10.3%	10.4%	11.6%	11.6%	10.3%
MI	7.1%	**89.5%***	**89.5%***	7.0%	7.2%	4.9%
MN	22.1%	23.1%	22.1%	7.8%	9.5%	11.0%
MO	**53.6%***	**52.7%***	43.6%	22.7%	19.6%	9.2%
MT	9.3%	11.5%	12.8%	14.0%	13.2%	13.9%
NC	0.4%	0.3%	0.3%	0.3%	0.2%	0.2%
ND	0.1%	0.1%	0.1%	0.1%	0.1%	0.1%
NE	**97.3%***	7.2%	7.7%	8.0%	7.5%	7.3%
NH	6.1%	8.2%	10.5%	13.1%	14.4%	9.9%
NJ	5.8%	6.0%	6.4%	7.2%	7.7%	8.5%
NM	1.0%	1.1%	1.1%	1.1%	1.1%	0.7%
NV	3.3%	3.2%	3.0%	3.1%	3.4%	3.6%
NY	31.8%	33.3%	34.9%	35.4%	35.9%	30.9%
OH	6.6%	7.2%	8.2%	8.3%	7.7%	7.6%
OK	5.7%	5.5%	5.5%	5.2%	6.5%	6.2%
OR	22.2%	23.1%	22.3%	21.5%	**52.8%***	27.6%
PA	5.3%	5.0%	5.5%	6.1%	6.6%	6.9%
SC	28.5%	33.5%	36.9%	40.9%	46.0%	49.4%
SD	0.0%	0.0%	0.0%	0.0%	0.0%	0.0%
TX	10.9%	11.7%	13.1%	14.5%	15.3%	16.1%
UT	34.0%	37.2%	41.8%	45.4%	**52.4%***	**55.2%***
VA	2.4%	2.1%	1.9%	2.1%	2.1%	2.9%
VT	19.9%	19.3%	20.2%	21.4%	21.9%	23.0%

© 2026 Zhang H et al. *JAMA Network Open*.

WA	12.9%	12.1%	11.1%	10.3%	10.0%	9.9%
WI	18.9%	19.1%	20.2%	21.7%	21.9%	16.5%
WV	10.8%	16.2%	22.6%	28.2%	21.0%	21.5%

[a] State-years corresponding to the cells marked with (*) were excluded from the subgroup analyses by race-ethnicity due to high missingness rates.

© 2026 Zhang H et al. *JAMA Network Open*.

eTable 5. Standardized Prevalence of Pediatric Diabetes per 1,000 Medicaid-enrolled Youths by Demographic Characteristics[a] (Allowing up to a 14-Day Coverage Gap)

Characteristics[b]	2016	2017	2018	2019	2020	2021	Relative increase	p[e]
Overall	2.76	2.79	2.87	2.99	2.87	3.01	9.1%	<0.001
Age								
0-6	0.45	0.47	0.48	0.50	0.50	0.51	13.3%	<0.001
7-12	2.11	2.15	2.19	2.29	2.30	2.42	14.7%	<0.001
13-18	5.90	5.98	6.03	6.13	6.08	6.49	10.0%	<0.001
Gender								
Male	2.49	2.56	2.60	2.68	2.72	2.91	16.9%	<0.001
Female	2.94	2.95	2.97	3.04	2.97	3.13	6.5%	<0.001
Race-ethnicity[d]								
Asian	1.47	1.45	1.45	1.52	1.54	1.75	19.0%	<0.001
Hispanic	2.11	2.17	2.26	2.35	2.32	2.58	22.3%	<0.001
Non-Hispanic Black	2.73	2.79	2.83	2.95	3.05	3.43	25.6%	<0.001
Non-Hispanic White	3.45	3.46	3.44	3.50	3.44	3.47	0.6%	0.1834
Other[c]	2.29	2.46	2.47	2.60	2.72	3.10	35.4%	<0.001
Census Region								
Midwest	3.08	3.14	3.14	3.25	3.11	3.19	3.8%	<0.001
Northeast	3.11	3.06	3.34	3.57	3.18	3.28	5.6%	<0.001
South	2.77	2.76	2.76	2.83	2.80	2.97	7.2%	<0.001
West	2.27	2.37	2.47	2.59	2.58	2.75	21.2%	<0.001
Residence								
Urban	2.61	2.65	2.69	2.75	2.77	2.96	13.4%	<0.001
Rural	3.17	3.22	3.23	3.28	3.22	3.33	5.1%	<0.001

[a] Diabetes cases were identified if there was one diabetes diagnosis and two antidiabetic drugs, or two diabetes diagnosis at least 30 days apart. The prevalence estimates were standardized with respect to age, gender, race, census region, and urban/rural residence. For each covariate of interest, prevalence rates were standardized to the distribution of the remaining covariates

© 2026 Zhang H et al. *JAMA Network Open*.

using the pooled study population across all study years as the standard population. Data from 12 states (AL, AR, FL, HI, MD, MS, OK, RI, TN, UT, WI, WY) and DC were excluded due to data quality issues.
[b] Pairwise deletion is used in the subgroup analysis
[c] The Other group includes American Indian and Alaska Native, Hawaiian/Pacific Islander, Multiracial, and other.
[d] States with the missingness rate above 50% were excluded from calculation.
[e] p value from Cochran–Armitage trend test.

© 2026 Zhang H et al. *JAMA Network Open*.

eTable 6. Standardized Prevalence of Type 1 Diabetes per 1,000 Medicaid-enrolled Youths by Demographic Characteristics[a] (Allowing up to a 14-Day Coverage Gap)

Characteristics[b]	2016	2017	2018	2019	2020	2021	Relative increase	p[e]
Overall	1.98	2.02	2.06	2.10	2.08	2.09	5.6%	<0.001
Age								
0-6	0.45	0.47	0.48	0.50	0.50	0.51	13.3%	<0.001
7-12	1.84	1.90	1.93	2.00	2.00	2.04	10.9%	<0.001
13-18	3.86	3.95	3.98	4.00	3.98	3.98	3.1%	<0.001
Gender								
Male	1.98	2.04	2.07	2.10	2.12	2.15	8.6%	<0.001
Female	1.99	2.04	2.06	2.10	2.07	2.07	4.0%	<0.001
Race-ethnicity[d]								
Asian	0.95	0.93	0.93	0.97	0.96	1.03	8.4%	<0.001
Hispanic	1.31	1.37	1.43	1.46	1.42	1.46	11.5%	<0.001
Non-Hispanic Black	1.71	1.79	1.84	1.91	1.96	2.05	19.9%	<0.001
Non-Hispanic White	2.89	2.93	2.92	2.97	2.94	2.91	0.7%	0.0104
Other[c]	1.35	1.37	1.40	1.58	1.57	1.80	33.3%	<0.001
Census Region								
Midwest	2.23	2.33	2.37	2.43	2.32	2.33	4.6%	<0.001
Northeast	2.37	2.34	2.36	2.36	2.42	2.42	2.4%	<0.001
South	1.83	1.85	1.86	1.91	1.89	1.90	4.0%	<0.001
West	1.68	1.76	1.83	1.87	1.86	1.88	12.2%	<0.001
Residence								
Urban	1.91	1.96	1.99	2.02	2.03	2.05	7.3%	<0.001
Rural	2.33	2.40	2.42	2.45	2.43	2.41	3.6%	<0.001

[a] Type 1 diabetes was defined if any of the following was met: 1) under 6 years old at diagnosis, 2) no antidiabetic drug use except for insulin, and 3) type 1 diabetes as the first two diagnosis. The prevalence estimates were standardized with respect to age, gender, race, census region, and urban/rural residence. For each covariate of interest, prevalence rates were standardized to

© 2026 Zhang H et al. *JAMA Network Open*.

the distribution of the remaining covariates using the pooled study population across all study years as the standard population. Data from 12 states (AL, AR, FL, HI, MD, MS, OK, RI, TN, UT, WI, WY) and DC were excluded due to data quality issues.
[b] Pairwise deletion is used in the subgroup analysis
[c] The Other group includes American Indian and Alaska Native, Hawaiian/Pacific Islander, Multiracial, and other.
[d] States with the missingness rate above 50% were excluded from calculation.
[e] p value from Cochran–Armitage trend test.

© 2026 Zhang H et al. *JAMA Network Open*.

eTable 7. Standardized Prevalence of Type 2 Diabetes per 1,000 Medicaid-enrolled Youths by Demographic Characteristics[a] (Allowing up to a 14-Day Coverage Gap)

Characteristics[b]	2016	2017	2018	2019	2020	2021	Relative increase	p[f]
Overall	0.78	0.77	0.81	0.89	0.80	0.92	17.9%	<0.001
Age[c]								
0-6	-	-	-	-	-			
7-12	0.27	0.26	0.26	0.29	0.30	0.38	40.7%	<0.001
13-18	2.04	2.03	2.05	2.12	2.10	2.51	23.0%	<0.001
Gender								
Male	0.51	0.52	0.54	0.58	0.60	0.76	49.0%	<0.001
Female	0.95	0.92	0.91	0.94	0.91	1.06	11.6%	<0.001
Race-ethnicity[e]								
Asian	0.52	0.52	0.52	0.55	0.58	0.72	38.5%	<0.001
Hispanic	0.80	0.80	0.83	0.89	0.90	1.12	40.0%	<0.001
Non-Hispanic Black	1.02	1.01	0.99	1.04	1.09	1.39	36.3%	<0.001
Non-Hispanic White	0.56	0.53	0.51	0.53	0.50	0.56	0.0%	0.0087
Other[d]	0.94	1.09	1.07	1.02	1.15	1.30	38.3%	<0.001
Census Region								
Midwest	0.85	0.80	0.77	0.82	0.78	0.86	1.6%	0.1712
Northeast	0.74	0.71	0.98	1.21	0.76	0.86	15.5%	<0.001
South	0.94	0.92	0.90	0.92	0.90	1.07	13.6%	<0.001
West	0.59	0.61	0.64	0.71	0.71	0.86	46.8%	<0.001
Residence								
Urban	0.70	0.70	0.70	0.74	0.74	0.91	30.0%	<0.001
Rural	0.84	0.81	0.80	0.83	0.80	0.92	9.2%	<0.001

[a] Type 2 diabetes was defined if none of the criteria for type 1 diabetes was met. The prevalence estimates were standardized with respect to age, gender, race, census region, and urban/rural residence. For each covariate of interest, prevalence rates were standardized to the distribution of the remaining covariates using the pooled study population across all study years as the standard population. Data from 12 states (AL, AR, FL, HI, MD, MS, OK, RI, TN, UT, WI, WY) and DC were excluded due to data quality issues.

© 2026 Zhang H et al. *JAMA Network Open*.

[b] Pairwise deletion is used in the subgroup analysis
[c] Pediatric diabetes for youths aged 0-6 were classified as type 2 based on our algorithm.
[d] The Other group includes American Indian and Alaska Native, Hawaiian/Pacific Islander, Multiracial, and other.
[e] States with the missingness rate above 50% were excluded from calculation.
[f] p value from Cochran–Armitage trend test.

© 2026 Zhang H et al. *JAMA Network Open*.

eFigure 1. Trend in the crude (unstandardized) prevalence of pediatric diabetes among Medicaid and Children's Health Insurance Program (CHIP) enrollees, 2016 -2021

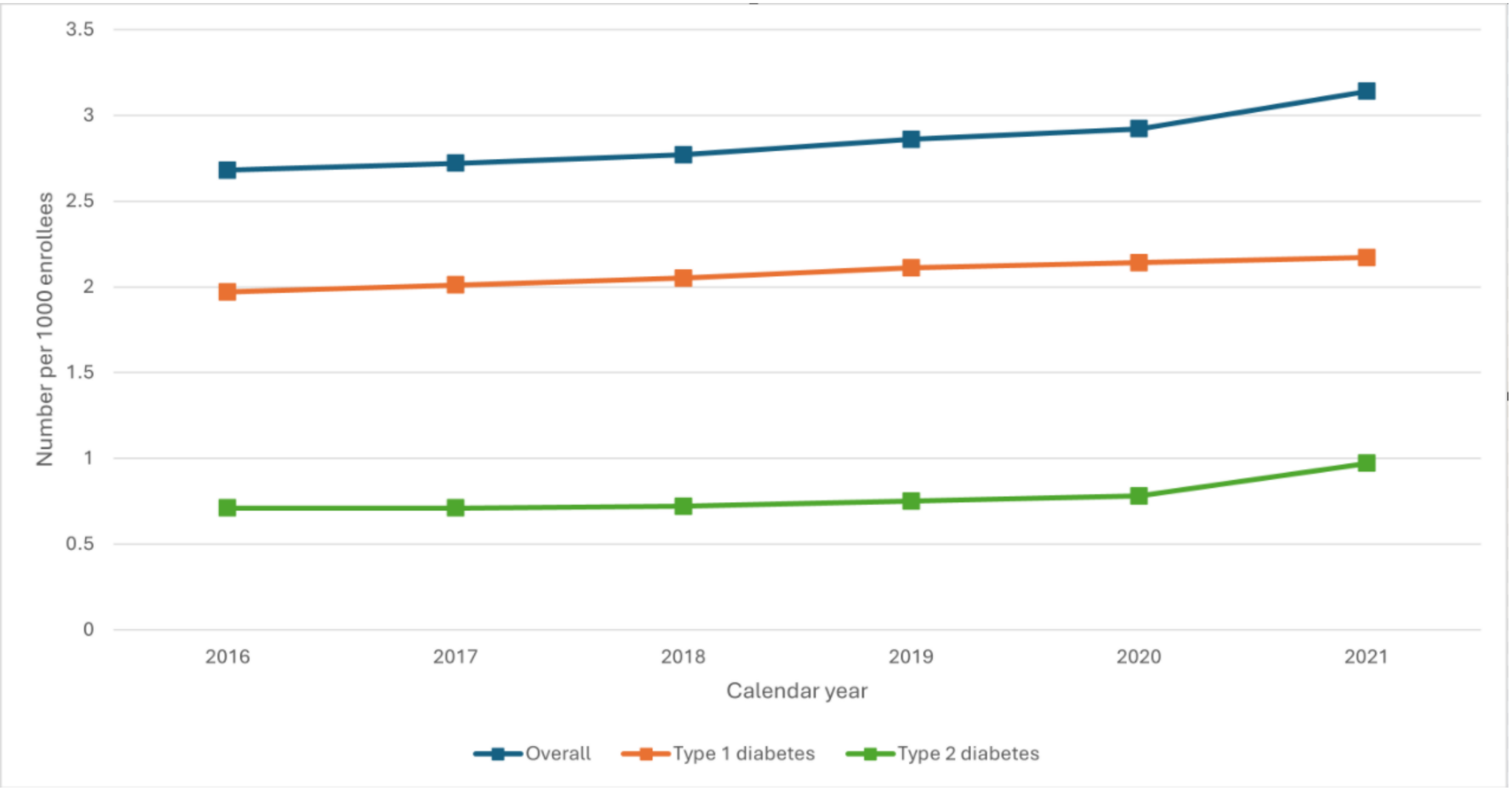

© 2026 Zhang H et al. *JAMA Network Open*.
