# Supplementary material for: Pediatric Diabetes Prevalence Among Medicaid Beneficiaries
Source: JAMA Netw Open. 2026 Feb 23;9(2):e2560507. doi: 10.1001/jamanetworkopen.2025.60507 (PMC12931460; doi:10.1001/jamanetworkopen.2025.60507)
Supplement: Supplement 2. — Data Sharing Statement [file jamanetwopen-e2560507-s002.pdf]

Data Sharing Statement

Zhang. Pediatric Diabetes Prevalence Among Medicaid Beneficiaries. *JAMA Netw Open*. Published February 23, 2026. doi:10.1001/jamanetworkopen.2025.60507

Data
Data available: No

Additional Information
Explanation for why data not available: The T-MSIS data was obtained through a Data Use Agreement between CMS and UAB, and we do not have permission to share these individual-level Medicaid data. However, researchers may apply to receive the data from ResDac. We will make available all our statistical code and the data dictionary to facilitate replication of our findings.
